# Correction: Scientific and engineering practices aligned with the NGSS in the performance of secondary stage physics teachers

**DOI:** 10.1371/journal.pone.0292771

**Published:** 2023-10-05

**Authors:** Mohammad Khair M. Al-Salamat

There are errors in the Funding section. The correct Funding statement is: This study was funded by the Deanship of Scientific Research at Taif University, Kingdom of Saudi Arabia (Grant No. 1-1442-34) awarded to Mohammad Khair Alsalamat. The funders had no role in the study design, data collection and analysis, the decision to publish, or the preparation of the manuscript.

## References

[1] Al-Salamat MKM (2022) Scientific and engineering practices aligned with the NGSS in the performance of secondary stage physics teachers. PLoS ONE 17(10): e0275158. 10.1371/journal.pone.0275158 36215309 PMC9550033

