# Supplementary material for: Scientific and engineering practices aligned with the NGSS in the performance of secondary stage physics teachers
Source: PLoS One. 2022 Oct 10;17(10):e0275158. doi: 10.1371/journal.pone.0275158 (PMC9550033; doi:10.1371/journal.pone.0275158)
Supplement: S1 File. Study instrument questionnaire — (PDF) [file pone.0275158.s001.pdf]

Dear physics teachers at the secondary stage,

Please respond to the following questionnaire. This questionnaire is for a study entitled (Scientific and Engineering Practices Aligned with the NGSS in the Performance of Secondary Stage Physics Teachers). In part one, please tick (✓) the answer that applies to you, and in Part Two, please respond to the items in the questionnaire by ticking (✓) the box that expresses your opinion.

Please bear in mind, that your answers will be used confidentially and for scientific research purposes only.

With all due respect and appreciation.

Researcher/ Mohammad Khair Salamat

Part One: Demographic Data

Name (optional): ....

Qualification ☐ Bachelor ☐ Postgraduate

Years of Experience ☐ less than 5 years ☐ From 5 to 10 years ☐ More than 10 years

Part Two: Questionnaire Items

Practice	No.	Indicator	Degree of practice				
			very high	high	medium	weak	Very weak
Asking questions and defining problems	1	I ask questions to explain the factors that created a certain physical phenomenon.					
	2	I request students to create surveys and questions related to various physical phenomena.					
	3	I specify the criteria and restrictions of designing physical problems to satisfactorily reach an accurate solution for these problems.					
	4	I encourage students to detect and identify physical problems.					
	5	I ask questions on the data reached to identify the factors influencing them.					
Developing and using models	1	I encourage students to develop ideas on various physical phenomena.					
	2	I request them to test hypotheses using the best mathematical and computational methods and to make decisions on what is or is not included in the physics model					
	3	I encourage them to develop causal interpretations for physical phenomenon of the real world.					
	4	I develop models to describe various physical phenomena.					
	5	I request students to explain physical phenomena using physical and mathematical terms.					
	6	I assess and review the teaching process via physical and mathematical acts.					
Planning and Carrying out Investigations	1	I request students to provide evidence which shows that change in physical phenomena depends on certain forces					
	2	I request them to check relations between components and causes of several physical phenomena					
	3	I evaluate the experimental design to prove that there are certain factors which affect any physical phenomenon					
	4	I request students to collect data to provide evidence on the mechanism of physical phenomena formation					
Analyzing and Interpreting Data	1	I request students to analyze and to interpret data to determine features of a certain physical phenomenon.					
	2	I request them to analyze exam data to identify similarities and differences between a number of solution designs to choose the best.					
	3	I request them to draw and interpret diagrams to describe certain physical correlations,					
	4	I encourage them to detect patterns and relations that permit using data to support a model or to interpret a result.					

Practice	No.	Indicator	Degree of practice				
			very high	high	medium	weak	Very weak
	5	I request them to analyze and interpret data to reach a result, provide evidence, or correct scientific solution.					
	6	I request them to present physical data in tables.					
	7	I request them to analyze data statistically.					
Using Mathematics and Computational Thinking	1	I request students to practice mathematical thinking in analyzing physical phenomena.					
	2	I request them to practice computational thinking in analyzing physical phenomena.					
	3	I encourage them to use mathematical examples to describe simple physical models.					
	4	I encourage them to complete the process of data collection and to analyze large amounts of them.					
	5	I request them to use mathematical examples to prove how certain factors influence the increase and decrease of certain physical features or phenomenon.					
Constructing Interpretations and Solution Design	1	I request students to adopt authentic and correct evidence to explain certain physical phenomena.					
	2	I request them to design various physical projects.					
	3	I request them to apply scientific law.					
	4	I request them to apply ideas of physics to construct interpretations for similarities and differences between things or different phenomena.					
	5	I request them to construct scientific interpretations based on evidence to show how certain external factors affect a physical phenomenon					
	6	I request them to apply principles and scientific theories to design a way to reduce the influence of certain factors on various physical phenomena.					
	7	I request students to construct a scientific interpretation based on evidence that describes how differences in certain features increase the possibility of survival of the physical phenomenon.					
Involvement with Proofs and Evidence	1	I encourage students to present assessments and justifications for constructing physical models and scientific explanations.					
	2	I request them to use the claims/hypotheses supported by evidence and experiment to clarify different physical systems.					
	3	I request them to compare different models and interpretations that show points of strength and weakness.					
	4	I request them to use verbal and written proofs to support or refute a model or an interpretation of a certain physical phenomenon.					

Practice	No.	Indicator	Degree of practice				
			very high	high	medium	weak	Very weak
	5	I encourage them to explain evaluations and evidence for each other.					
Obtaining, Evaluating, and Communicating Information	1	I request students to distinguish between observation and induction, request and evidence, proof and interpretation.					
	2	I request them to use diverse means of communication patterns such as diagrams, models, and equations.					
	3	I request them to read scientific physics texts and interpret them					
	4	I request them to produce a scientific text in order to develop and explain a physics model.					
	5	I request students to collect data in order to describe a certain physical phenomenon.					

Thank you
