# Supplementary material for: Scientific and engineering practices aligned with the NGSS in the performance of secondary stage physics teachers
Source: PLoS One. 2022 Oct 10;17(10):e0275158. doi: 10.1371/journal.pone.0275158 (PMC9550033; doi:10.1371/journal.pone.0275158)
Supplement: S2 File. Study instrument interview — (PDF) [file pone.0275158.s002.pdf]

Dear Education Supervisor,

Based on your supervision of physics teachers at the secondary stage, please answer the following questions:

Q1. To what degree do physics teachers at the secondary stage practice "Asking questions and defining problems"? And what are the reasons for that?

Q2. To what degree do physics teachers at the secondary stage practice "Developing and using models"? And what are the reasons for that?

Q3. To what degree do physics teachers at the secondary stage "Planning and carrying out investigations"? And what are the reasons for that?

Q4. To what degree do physics teachers at the secondary stage practice "Analyzing and interpreting data"? And what are the reasons for that?

Q5. To what degree do physics teachers at the secondary stage practice "Using mathematics and computational thinking"? And what are the reasons for that?

Q6. To what degree do physics teachers at the secondary stage practice "Constructing interpretations and solution designs"? And what are the reasons for that?

Q7. To what degree do physics teachers at the secondary stage practice "Involvement with proofs and evidence"? And what are the reasons for that?

Q8. To what degree do physics teachers at the secondary stage practice "Obtaining, evaluating, and communicating information"? And what are the reasons for that?

Thank you
