# Supplementary material for: Scientific and engineering practices aligned with the NGSS in the performance of secondary stage physics teachers
Source: PLoS One. 2022 Oct 10;17(10):e0275158. doi: 10.1371/journal.pone.0275158 (PMC9550033; doi:10.1371/journal.pone.0275158)
Supplement: Attachment — Submitted filename: PONE-D-21-34137_reviewer.pdf with comments.pdf [file pone.0275158.s005.pdf]

PLOS ONE

Scientific and Engineering Practices Aligned with NGSS Standards in the Performance of Secondary Stage Physics Teachers

--Manuscript Draft--

**Manuscript Number:**	PONE-D-21-34137
**Article Type:**	Research Article
**Full Title:**	Scientific and Engineering Practices Aligned with NGSS Standards in the Performance of Secondary Stage Physics Teachers
**Short Title:**	Scientific and Engineering Practices Aligned with NGSS Standards in the Performance of Physics Teachers
**Corresponding Author:**	Mohammad Khair Alsalamat<br>Taif University<br>Taif, SAUDI ARABIA
**Keywords:**	Scientific and Engineering Practices - NGSS - Secondary Stage -Physics Teachers
**Abstract:**	The study aimed to unravel the degree of availability of scientific and engineering practices that comply with NGSS standards in the performance of physics teachers at the secondary stage in Taif city. The study adopted a mixed method in applying a questionnaire that comprised (44) items. It was applied to (49) teachers who were randomly selected, in addition to (6) education supervisors who were purposively selected. The findings revealed that the following five practices: "asking questions and defining problems", "obtaining, evaluating, and communicating information", besides "Planning and carrying out investigations "Analyzing and interpreting data", and finally "involvement with proofs and evidences", all rated medium. The three practices: "interpretation and design solutions", "developing and using models", "Using mathematics and computational thinking" all rated weak. Data analysis of education supervisors' interviews revealed that their opinions were concordant to an extent with the questionnaire findings. The results also revealed that the practices of postgraduate teachers were better than those of teachers with B.As, who, irrespective of years of experience, got the same results. It might be noted that teachers of physics need to reconsider their teaching practices to aligned with NGSS.
**Order of Authors:**	Mohammad Khair Alsalamat
**Additional Information:**	
**Question**	**Response**
**Financial Disclosure**<br><br>Enter a financial disclosure statement that describes the sources of funding for the work included in this submission. Review the submission guidelines for detailed requirements. View published research articles from *PLOS ONE* for specific examples.<br><br>This statement is required for submission and **will appear in the published article** if the submission is accepted. Please make sure it is accurate.	yes<br>Taif University<br>The funders had no role in study design, data collection and analysis, decision to publish, or preparation of the manuscript.

**Unfunded studies**<br>Enter: *The author(s) received no specific funding for this work.*<br><br>**Funded studies**<br>Enter a statement with the following details:<br>• Initials of the authors who received each award<br>• Grant numbers awarded to each author<br>• The full name of each funder<br>• URL of each funder website<br>• Did the sponsors or funders play any role in the study design, data collection and analysis, decision to publish, or preparation of the manuscript?<br>• **NO** - Include this sentence at the end of your statement: *The funders had no role in study design, data collection and analysis, decision to publish, or preparation of the manuscript.*<br>• **YES** - Specify the role(s) played.<br><br>* typeset	
**Competing Interests**<br><br>Use the instructions below to enter a competing interest statement for this submission. On behalf of all authors, disclose any competing interests that could be perceived to bias this work—acknowledging all financial support and any other relevant financial or non-financial competing interests.<br><br>This statement is required for submission and **will appear in the published article** if the submission is accepted. Please make sure it is accurate and that any funding sources listed in your Funding Information later in the submission form are also declared in your Financial Disclosure statement.<br><br>View published research articles from *PLOS ONE* for specific examples.	The authors have declared that no competing interests exist.

**NO authors have competing interests**<br><br>Enter: *The authors have declared that no competing interests exist.*<br><br>**Authors with competing interests**<br><br>Enter competing interest details beginning with this statement:<br><br>*I have read the journal's policy and the authors of this manuscript have the following competing interests: [insert competing interests here]*<br><br>* typeset	
**Ethics Statement**<br><br>Enter an ethics statement for this submission. This statement is required if the study involved:<br><br>• Human participants<br>• Human specimens or tissue<br>• Vertebrate animals or cephalopods<br>• Vertebrate embryos or tissues<br>• Field research<br><br>Write "N/A" if the submission does not require an ethics statement.<br><br>General guidance is provided below. Consult the submission guidelines for detailed instructions. **Make sure that all information entered here is included in the Methods section of the manuscript.**	The study adopted the mixed approach. Quantitative data were collected from (49) Secondary Stage Physics Teachers in Taif city, randomly selected, first semester 2021/2022. The qualitative data were selected from (6) physics supervisors who agreed to participate. Confidentiality and independence were made clear to participants from the onset of collecting the data. All participants (Teachers and supervisors) provided consent to participate in the study.

Powered by Editorial Manager® and ProduXion Manager® from Aries Systems Corporation

**Format for specific study types**<br><br>**Human Subject Research (involving human participants and/or tissue)**<br>• Give the name of the institutional review board or ethics committee that approved the study<br>• Include the approval number and/or a statement indicating approval of this research<br>• Indicate the form of consent obtained (written/oral) or the reason that consent was not obtained (e.g. the data were analyzed anonymously)<br><br>**Animal Research (involving vertebrate animals, embryos or tissues)**<br>• Provide the name of the Institutional Animal Care and Use Committee (IACUC) or other relevant ethics board that reviewed the study protocol, and indicate whether they approved this research or granted a formal waiver of ethical approval<br>• Include an approval number if one was obtained<br>• If the study involved *non-human primates*, add *additional details* about animal welfare and steps taken to ameliorate suffering<br>• If anesthesia, euthanasia, or any kind of animal sacrifice is part of the study, include briefly which substances and/or methods were applied<br><br>**Field Research**<br><br>Include the following details if this study involves the collection of plant, animal, or other materials from a natural setting:<br>• Field permit number<br>• Name of the institution or relevant body that granted permission	
**Data Availability**<br><br>Authors are required to make all data underlying the findings described fully available, without restriction, and from the time of publication. PLOS allows rare exceptions to address legal and ethical concerns. See the PLOS Data Policy and FAQ for detailed information.	Yes - all data are fully available without restriction

A Data Availability Statement describing where the data can be found is required at submission. Your answers to this question constitute the Data Availability Statement and **will be published in the article**, if accepted.<br><br>**Important:** Stating 'data available on request from the author' is not sufficient. If your data are only available upon request, select 'No' for the first question and explain your exceptional situation in the text box.<br><br>Do the authors confirm that all data underlying the findings described in their manuscript are fully available without restriction?	
**Describe where the data may be found in full sentences. If you are copying our sample text, replace any instances of XXX with the appropriate details.**<br><br>• If the data are **held or will be held in a public repository**, include URLs, accession numbers or DOIs. If this information will only be available after acceptance, indicate this by ticking the box below. For example: *All XXX files are available from the XXX database (accession number(s) XXX, XXX.*).<br>• If the data are all contained **within the manuscript and/or Supporting Information files**, enter the following: *All relevant data are within the manuscript and its Supporting Information files.*<br>• If neither of these applies but you are able to provide **details of access elsewhere**, with or without limitations, please do so. For example:<br><br>*Data cannot be shared publicly because of [XXX]. Data are available from the XXX Institutional Data Access / Ethics Committee (contact via XXX) for researchers who meet the criteria for access to confidential data.*<br><br>*The data underlying the results presented in the study are available from (include the name of the third party*	xxx

Powered by Editorial Manager® and ProduXion Manager® from Aries Systems Corporation

and contact information or URL).<br>• This text is appropriate if the data are owned by a third party and authors do not have permission to share the data.<br><br>* typeset	
Additional data availability information:	

Powered by Editorial Manager® and ProduXion Manager® from Aries Systems Corporation

no author name Click here to access/download;Manuscript;NGSS reserch Moh.doc

Scientific and Engineering Practices Aligned with NGSS Standards in the Performance of Secondary Stage Physics Teachers

Mohammad Khair Al-Salamat[1]

m.alsalamat@tu.edu.sa

[1]Taif University -Department of Curriculum and Educational Technologies

ABSTRACT

The study aimed to unravel the degree of availability of scientific and engineering practices that comply with NGSS standards in the performance of physics teachers at the secondary stage in Taif city. The study adopted a mixed method in applying a questionnaire that comprised (44) items. It was applied to (49) teachers who were randomly selected, in addition to (6) education supervisors who were purposively selected. The findings revealed that the following five practices: "asking questions and defining problems", "obtaining, evaluating, and communicating information", besides "Planning and carrying out investigations "Analyzing and interpreting data", and finally "involvement with proofs and evidences", all rated medium. The three practices: "interpretation and design solutions", "developing and using models", "Using mathematics and computational thinking" all rated weak. Data analysis of education supervisors' interviews revealed that their opinions were concordant to an extent with the questionnaire findings. The results also revealed that the practices of postgraduate teachers were better than those of teachers with B.As, who, irrespective of years of experience, got the same results. It might be noted that teachers of physics need to reconsider their teaching practices to aligned with NGSS.

KEYWORDS

Scientific and Engineering Practices - NGSS - Secondary Stage -Physics Teachers

Introduction and theoretical background

NGSS is one of the foremost standards in the field of education in general and that of evaluation in particular, due to the interaction it received from those concerned with it. Education and teaching institutions adopted such standards in order to develop their education systems because of the active role such standards play to accurately identify the

targeted results of the education process. Many institutions catered for developing the field of teaching and learning science, relying on such standards.

Among those are: National Science Teaching Association (NSTA), National Service Framework (NSF), American Association for the Advancement of Sciences (AAAS), and National Science Teaching Association (NSTA). Many projects of developing teaching science appeared such as: Specialized Technical Services (STS) for reforming science curricula in light of interaction between science, technology, and society, National Standards for Teaching Sciences, 2061 project, and California Standards for Science curriculum.

In addition to the preceding projects, a new document for science standards was developed in 2011 by twenty-six American States under the auspices of the National Research Council (NRC), side by side with a team of (41) members. The document dealt with science for all stages, from kindergarten till twelfth level (Achieve, (K-12), 2013), producing NGSS which is the most updated directives for reforming and developing teaching and learning science. The NGSS was designed to suit all classes from kindergarten stage to secondary (K-12). It also anticipates the future performance of students and rests on three major dimensions represented in: main ideas, scientific and engineering practices, and comprehensive concepts (Tyler & Diranna, 2018 ; Duschl & Bybee, 2014).

Because students' objectives for learning sciences in general and physics in particular have changed, science teachers of all levels need to adapt themselves to these new standards in order to achieve an actual change in their teaching practices. Therefore, educators see that teachers' qualifying programs should be developed to make teachers concentrate more on actual practice and to help them amend their practices. They should also be provided with purposeful training chances that support NGSS, and to exert an effort to make teachers change their attitude towards the practices dictated by NGSS which largely contributes to the achievement of required objectives (Severance et al., 2016 ; Bielik et al, 2021).

Among the most important objectives of NGSS and science teaching are: to make students behave and practice in a scholarly manner to gain skills of engineering design which enable them to conduct research and to solve problems they encounter throughout science study or in their actual life. Therefore, those in charge of NGSS adopted the term scientific practices instead of science processes because it combines practices and skills of whoever teaches science, besides skills of the engineer who solves problems (Harris et al., 2017). This has been

done to ensure students' obtaining the correct and real mechanism by which scientific research is conducted. It is the mechanism through which scientists, like Einstein, were able to come up to their discoveries (NRC, 2012 ; Boesdorfer & Staude, 2016 ; Bielik et al., 2021).

By reviewing the official website of NGSS and the various studies that tackled scientific and engineering practices from several perspectives, with regard to measuring their range of actualizing teaching, detecting attitudes of teachers and the concerned, or to unraveling the range of students' possessing such standards at the numerous stages of learning, as discussed in the following studies: (NGSS, 2013 ; Hang & Srisawasdi, 2021 ; Pruitt, 2015 ; Malkawi & Rababah, 2018 ; Boesdorfer & Staude, 2016 ; Alshyab, 2019 ; Aboathrah, 2019 ; Stuart et al., 2021). It was made clear that there were eight practices: **First**, "asking questions (science) defining problems (engineering)" by which student's mind and thinking are triggered through brainstorming that they use in asking questions to identify the problem. **Second** practice, "developing and using models" here, the student puts a mental or practical application by which he demonstrates the phenomenon or the problem, subject of the study, and practices physics by constructing a model that helps in interpretation and prediction. He, then, is engineeringly directed via that model by which he analyzes the surrounding systems to come up to probable solutions. **Third** practice, Planning and carrying out investigations where the student is placed in a position that enables him to practice observation, to analyze, to conduct surveys in order to propose and test a hypothesis. **Fourth** practice "Analyzing and interpreting data" where the students is instructed to analyze the data and information he collected from previous practices, using diagrams, statistical analysis, data processing and interpretation. **Fifth** practice "involvement with pretexts, proofs and evidences" by which the student extracts pretexts and proofs to identify points of weakness and strength in order to choose the best interpretation for the phenomena, and to criticize and evaluate opinions of others. **Sixth** practice, "interpretations and solutions design" by which the student constructs theories to interpret such a phenomenon. He uses systematic solutions for the problem choosing the primal one from the proposed designs. **Seventh** practice, "obtaining, evaluating and communicating information" where the student reads scientific texts and explains them, with an aim of developing, interpreting, evaluating information sources, to secure validity. **Eighth** practice, "Using mathematics and computational thinking" where the student uses various mathematical and engineering skills, as math and computational thinking constitute a significant part for science and engineering. In such a case scientific models that illustrate the phenomena are presented, This presentation might be arithmetical or symbolic and provides a scientific or logical interpretation for the diverse models.

Pursuant to curricula reform, movement in general and NGSS in particular, constant comprehensive updating and developing of the teaching-education system became necessary, specifically developing performance of physics teachers. Such a development provides the teacher with necessary competences which cope with those standards. Because previous studies didn't directly and specifically tackle such practices of the teacher of physics, the current one attempts to unveil the degree of availability of scientific and engineering practices that get along with NGSS regarding the performance of physics teacher of the secondary stage.

Statement of the problem

Educators and those concerned with NGSS see that physics teachers should possess such standards in general and scientific and engineering practices in particular. (Hang & Srisawasdi, 2021 ; Bielik et al., 2021 ; Dalvi et al., 2021 ; Stuart et al., 2021 ; Malkawi & Rababah, 2018 ; Duschl & Bybee, 2014 ; Bybee, 2014 ; Tuttle et al., 2016 ; Brownstein & Horvath ,2016 ; Daisly, 2016 ; Kawasaki, 2015 ; Fulcher, 2014). Therefore, the more practice they perform on such standards, the better their teaching performance will be and that will sequentially be reflected in the performance of their students, and in achieving objectives of teaching science in a better way. Despite the latest reforms related to the manner of involving students with physics, yet there are still big challenges that confront teachers who attempt to constantly apply science practices in class (Zhang & Wong, 2021). Thus, the current study endeavors to unravel the degree of availability of scientific and engineering practices that cope with NGSS, regarding performance of physics teachers, through answering the following two questions:

1- What is the degree of availability pertaining scientific and engineering practices that cope with NGSS in the performance of physics teachers at the secondary stage?
2- Does that degree of availability in performance of those teachers vary in accordance with qualification, or years of experience?

Significance of the study

This study tackles NGSS, particularly the scientific and engineering practices, one of the modern trends and criteria that should be cared for in teaching physics. The study might also make those in charge of preparing physics teachers concentrate on preparing pre-or-in-service programs of scientific and engineering practices. It might also be beneficial for education supervisors through acquainting them with the method of assessing teachers, using a list of scientific and engineering practices that they came up to. In addition, the study might also identify training needs for such teachers.

Terms of the study

NGSS: These are American standards that provide a new vision for teaching science in America. They comprise three integrated dimensions: major ideas, scientific and engineering practices, and common concepts (Kawasaki & William, 2020). Procedurally, it is a set of standards associated with the process of teaching and learning physics at the secondary stage. They determine what students of physics should have learnt focusing on the aforementioned three dimensions. The current study particularly focuses on the scientific and engineering practices needed for teachers of physics at the stage of concern.

Scientific and engineering practices: It is the second applicable dimension of NGSS which incorporates eight practices related to science and engineering. These are: "asking questions and defining problems", "developing and using models", "Planning and carrying out investigations", "analyzing and interpreting data", "Using mathematics and computational thinking", "constructing interpretations and designing solutions", "involvement with pretexts, proofs, and evidences", and finally "obtaining, evaluating, and communicating information" (NGSS, 2013 ; Alebous, 2021 ; Jimenez-Liso et al., 2021). The study adopted these eight practices and the items contained therein which amounted to (44) students of physics at the secondary stage. These were measured through individuals’ responses to the two instruments of the study set for this purpose.

Study limits and limitations

The study was confined to measuring scientific and engineering practices (contained in the instruments), being one of the three major dimensions of NGSS. It was also limited to a sample of physics teachers at the secondary stage, besides education supervisors in Taif city, first semester- 2021/2022. Results were determined by the range of validity and reliability of the study.

Methodology

Study approach and participants

The study adopted the mixed approach. Quantitative data were collected from (49) Secondary Stage Physics Teachers in Taif city, randomly selected, first semester 2021/2022. The qualitative data were selected from (6) physics supervisors who agreed to participate. Confidentiality and independence were made clear to participants

from the onset of collecting the data. All participants (Teachers and supervisors) provided consent to participate in the study.

Study instruments

To achieve its objectives, the study used the following two instruments:

- **Questionnaire**

After determining objectives of the questionnaire, the researcher checked the official website of NGSS (https:www.nextgenescience.org), modern education literatures, besides surveying scientific and engineering practices relevant to teachers of physics at the secondary stage as presented in (Malkawi & Rababah, 2018 ; Hang & Srisawasdi, 2021 ; Alshyab, 2019 ; Aboathrah, 2019 ; Brownstein & Horvath, 2016 ; Qablan, 2016 ; Fulcher, 2014 ; Stuart et al., 2021 ; Duschl & Bybee, 2014 ; Kawasaki, 2015), and the opinion of some specialists in teaching sciences.

The questionnaire comprised (8) practices and (44) items. Responses were measured in accordance to Likert's five-point scale (very high, high, medium, weak, very weak). The questionnaire was verified for validity and reliability by applying it to an exploratory sample of (15) teachers of physics. The criterion of judgement was as follows: if the mean is less than (1.36), the degree of availability is "very weak"; (1.80-2.60) weak; (2.60-3.40); medium; (3.40-4.20), high; (4.20-more) very high.

- **Interview**

Eight open-ended questions were prepared; each one echoes the viewpoint of an education supervisor regarding the degree of availability found in the practices of teachers of physics at the secondary stage. The questions were presented to specialists in science for their opinion. To ensure validity and reliability of the interview, participating supervisors were encouraged to give correct answers and enough time was given for recording those interviews in order to be analyzed and interpreted in depth. The interpretation of the supervisor of concern was presented to him to make sure that it echoed what he meant. To ensure independence and validity of the interview, more details were given to the supervisors regarding study sample, mechanism of choice, besides interview preparation and application.

Study results

First question: " What is the degree of availability pertaining scientific and engineering practices that cope with NGSS in the performance of physics teachers at the secondary stage?"

In answering this question, means and standard deviations of teachers' responses and supervisors' interviews were calculated as presented in table 1.

Table 1. Means and standard deviations for teachers' responses pertaining scientific and engineering practices

Scientific and engineering practices	Mean	Standard deviation	Degree of availability	Rank
Asking questions and defining problems	3.24	0.40	Medium	1
Developing and using models	2.51	0.60	Weak	7
Planning and carrying out investigations	3.03	0.65	Medium	3
Analyzing and interpreting data	2.74	0.51	Medium	4
Using mathematics and computational thinking	2.29	0.66	Weak	8
Constructing interpretations and solution designs	2.52	0.51	Weak	6
Involvement with pretexts, proofs, and evidence.	2.72	0.68	Medium	5
Obtaining evaluating, and communicating information	3.15	0.33	Medium	2
**General mean**	**2.69**	**0.41**	**Medium**	

Table 1 shows that the general mean of degree of availability of teachers' practices was "medium", (2.69). Five practices ranked medium; these are: (asking questions and defining problems) rated first, followed by "obtaining, evaluating and communicating information" second, "Planning and carrying out investigations", third, "Analyzing and interpreting data", fourth; "involvement with pretexts, evidences, and proofs", fifth. Three practices ranked "weak". Those are: "interpretation construction and solution design" rated sixth, "developing and using models", seventh, and finally "Using mathematics and computational thinking" eighth.

Thus, one can notice that physics teachers possess a limited awareness of the significance of those practices and that was reflected in their performance. This might be attributed to some hurdles such as: absence of some components of teachers' preparing programs during the pre-or in-service period, teaching and management load, and

shortage of lab equipment in many schools as that impacted all practices of teachers. This result agrees with those of (Hang & Srisawasdi, 2021 ; Aboathrah, 2019 ; Alshyab, 2019 ; Malkawi & Rababah, 2018 ; Kawasaki, 2015).

The item of teachers' scientific and engineering practices might be elaborated on as follows:

- **Asking questions and defining problems**

Table 2. presents results pertaining this type of practice.

Table 2. Means and deviations of the degree of availability regarding the practice of asking questions, defining problems and their indications.

No.	Items	Mean	Standard deviation	Degree of availability	Rank
1-	I ask questions to explain the factors that created a certain physical phenomenon	3.43	0.0.91	High	3
2-	I request students to create surveys and questions pertaining various physical phenomena	2.98	0.92	Medium	4
3-	I specify the criteria and restrictions of designing physical problems to satisfactorily secure an accurate solution for them	2.80	0.89	Medium	5
4-	I encourage students to detect and identify physical problems	3.49	0.84	High	2
5-	I ask questions on the data achieved to identify the factors influencing them	3.53	0.82	High	1
	**General mean**	**3.24**	**0.40**	**Medium**	

Table 2 shows that the general mean was (3.24) indicating a medium degree of availability. Three items ranked "high"; they are as follows: (no.5) rated first, (no.4) second, and (no.1) third, while two items ranked "medium"; (no.2) rated fourth and (no.3) rated fifth. Such results reveal that physics teachers somewhat care about asking questions to check the range of student's understanding of the data gained, besides correlating what he learnt to physical phenomena and problems he encounters in his life. The table also reveals that physics teachers tackle physical problems taken from the student's life asking him to identify them and suggest the proper restrictions and criteria that enable him to choose the propitious solution. Such a result agrees with the outcome of the following studies: (Malkawi & Rababah, 2018 ; Harris et al., 2017 ; Tuttle et al., 2016 ; Daisly, 2016 ; Kawasaki, 2015 ; Alshyab, 2019 ; Aboathrah, 2019 ; Qablan, 2016 ; Brownstein & Horvath , 2016).

Results of supervisors' interviews revealed that physics teachers ask questions in an acceptable way. They attributed such a thing to teachers' training because physical phenomena perplex students. All supervisors agreed that teachers' performance was lower than what was required from them. Such a thing negatively affected students' skills in defining scientific problems due to the improper preparation of the teachers in this domain. It is noteworthy to tell that teacher preparing programs, whether pre-or in-service, were mostly theoretical. The supervisors' attitudes agree with results of the following studies: (Alshyab, 2019 ; Aboathrah, 2019 ; Qablan, 2016 ; Brownstein & Horvath , 2016).

- **Developing and using models**

Table 3 presents results pertaining this practice

Table 3. Means and standard deviations regarding degree of availability of this practice and its indications.

No.	Items	Mean	Standard deviation	Degree of availability	Rank
1-	I encourage students to develop ideas on various physical phenomena	2.43	1.00	Weak	5
2-	I request them to test hypotheses using the best mathematical and computational methods and to take decisions on the included or non-included in physics model	2.49	1.17	Weak	4
3-	I encourage them to develop causal interpretations for physical phenomenon of the real world	2.35	1.16	Weak	6
4-	I develop models to describe various physical phenomena	2.55	1.21	Weak	7
5-	I request students to explain physical phenomena using physical and mathematical terms	2.63	1.17	Medium	3
6-	I assess and review the teaching process via physical and mathematical acts	2.61	1.02	Medium	1
	**General mean**	**2.51**	**0.60**	**Weak**	**2**

Table 3 shows that the general mean for the availability ranked "medium", (2.51); two items ranked "medium", (no.5) and (no.6): the former rated first and the latter second. The other remaining four items ranked "weak". Although physics necessitates using scientific predictions for the various phenomena which obligate using models, yet the findings reveal that many teachers still don't have such practice. This might be caused by teachers pre-and

in-service programs that didn’t help them acquire the skill of model developing, in addition to burdens assigned for them regarding teaching load, time constraints and long school curriculum.

The analysis of supervisors’ interviews revealed an agreement between them and teachers’ viewpoints. Two supervisors pointed out that physics teachers are clearly weak with regard to developing and using the models in teaching; most of them still use traditional methods of teaching that depend on instructing. They also revealed that long science curriculum, besides teaching burdens and administrative works teachers are required to do are obstacles in the way of using and developing models. Despite that, the supervisors noted that physics teachers are able to understand the mechanism of how their students think. Consequently, they ask them to correlate relations between phenomena and diversified systems.

Two other supervisors pointed out that some teachers showed an improvement in using modern teaching strategies in general and models in particular as a result of using higher studies whose syllabi tackle such issues.

Such a result agrees with the findings of the following studies (Hang & Srisawasdi, 2021 ; Alshyab, 2019 ; Aboathrah, 2019) in which practicing and developing models ranked "low", but differs from the findings of the following studies (Harris et al., 2017 ; Brownstein & Horvath, 2016 ; Tuttle et al., 2016 ; Daisly, 2016 ; Kawasaki, 2015) which pointed out that teachers’ performance pertaining NGSS ranked "good".

- **Planning and carrying out investigations**

Table 4 presents results pertaining this practice.

Table 4. Means and standard deviations regarding degree of availability of Planning and carrying out investigations with their indications.

No.	Items	Mean	Standard deviation	Degree of availability	Rank
1-	I request students to provide evidences which show that change on physical phenomena depends on certain forces	3.08	1.03	Medium	2
2-	I request them to check relations between components and causes of several physical phenomena	2.90	1.17	Medium	4
3-	I evaluate the experimental design to prove	2.98	0.94	Medium	3

	that there are certain factors which affect any physical phenomenon				
4-	I request students to collect data to provide evidence on the mechanism of physical phenomena formation	3.16	0.65	Medium	1
	**General mean**	**3.03**	**0.65**	**Medium**	

Table 4 shows that the general mean of this practice ranked "medium" (3.03). Although nature of subject matter of physics requires experimentation and scientific report writing that needs activation of surveying practice, yet teachers revealed that their practice of Planning and carrying out investigations was medium. Such a thing indicates that the practical aspect of teaching physics was still below the required level.

It also shows that teachers. in preparing their programs, have to focus more on the practical side and to explain why scientific experiments are important for teaching physics. In addition, it is important for the student to acquire skills of data collecting via sound scientific methods and to use that correctly to explain his opinion and the occurrence of any scientific phenomenon. (Duschl & Bybee, 2014) see that teachers should ask their students to conduct scientific investigations and to provide pretexts and evidences of the results they came up to.

Interviews results revealed that all supervisors' sample confirmed that despite their conviction of the importance of planning for teaching sciences, yet the majority still use ready plans. The supervisors, thus, notice a difference between what is written in the daily or semester plan and the actual performance of the teacher in class room.

They also assure that teachers' levels in the domain of planning can't be judged via records available with them, so they ascertained that programs and incentives to practice good planning and to perfect hem are essential.

The supervisors added that many teachers don't understand the concept of surveying; consequently, the teaching method they use is the traditional one, but very few of the distinguished teachers use teaching strategies based on surveying. As a result, students' level of surveying skills is generally weak.

Findings of the questionnaire and interviews that the researcher came up to agree with results of the following studies (Alshyab, 2019 ; Aboathrah, 2019 ; Harris et al., 2017 ; Brownstein & Horvath, 2016 ; Daisly, 2016 ; Kawasaki, 2015). which revealed that teachers' performance with regard to NGSS was medium or good.

- **Analyzing and interpreting data**

Table 5 shows results pertaining this practice.

Table 5. Means and standard deviations of degree of availability pertaining practice of Analyzing and interpreting data with their indications.

No.	Items	Mean	Standard deviation	Degree of availability	Rank
1-	I request students to analyze and to interpret data to determine features of a certain physical phenomenon	2.92	1.19	Medium	3
2-	I request them to analyze exam data to identify similarities and differences between a number of solution designs to choose the best	1.90	1.04	Weak	7
3-	I request them to draw and interpret diagrams to describe certain physical correlations	2.96	1.21	Medium	2
4-	I encourage them to detect patterns and relations that permit using data to support a model or to interpret a result	2.96	1.21	Medium	6
5-	I request them to analyze and interpret data to come up to a result to provide evidence, or correct scientific solution	2.77	1.21	Medium	5
6-	I request them to present physical data in tables	3.06	1.03	Medium	1
7-	I request them to analyze data statistically	2.90	1.18	Medium	4
	**General mean**	**2.74**	**0.51**	**Medium**	

Table 5 shows that the general mean for the availability of this practice ranked "medium" (2.74). All items ranked medium except for (no. 2) which ranked weak, rating last.

Such a result shows that some teachers still don't realize the significance of students' possessing the practice of analyzing and interpreting scientific data, besides its significance in solving problems the students encounter in their real life.

In fact, teachers need to consider its significance while preparing programs. Without this analysis, correlations, causes, system components, and understanding the physical phenomenon propitiously, could never be identified. Moreover, understanding many topics of physics requires comparisons between them and other phenomena. This entails analyzing every topic or phenomenon separately then make the required comparison. It is important that teachers should have the desire and motivation to acquaint their students with the scientific evidence on which they rely. The best way of getting such evidence is to analyze the data collected from different scientific phenomena. By analyzing results related to data analysis and interpretation of supervisors' interview, it was noted that they all confirmed that physics teachers practice this skill, but not to the required level. They attributed this to the traditional teaching method teachers still use, in addition to lack of teacher-preparing programs in general and physics teachers in particular; they still rely on ready-study plans and analyses, added to that the large number of students in classes besides teaching and administration burdens.

Such a result agrees with those of the following studies: (Hang & Srisawasdi, 2021 ; Alshyab, 2019 ; Aboathrah, 2019 ; Qablan, 2016) which pointed out that teachers' performance ranked medium.

- **Using mathematics and computational thinking.**

Table 6 presents results pertaining this practice.

Table 6. Means and standard deviations of degree of availability pertaining the Using mathematics and computational thinking with their indications.

No.	Items	Mean	Standard deviation	Degree of availability	Rank
1-	I request students to practice mathematical thinking in analyzing physical phenomena	2.26	1.27	Weak	4
2-	I request them to practice computational thinking in analyzing physical phenomena	2.09	1.19	Weak	5
3-	I encourage them to use mathematical examples to describe simple physical models.	2.32	1.28	Weak	2
4-	I encourage them to complete the process of data collection and to analyze large amounts of them	2.27	1.30	Weak	3
5-	I request them to use mathematical examples to prove how certain factors influence the increase and decrease of certain physical features or	2.49	1.18	Weak	1

phenomenon				
**General mean**	**2.29**	**0.66**	**Weak**	

Table 6 shows that the general mean for the availability of this practice ranked "weak", (2.29) which implies that all indicators were weak. Such a result might be attributed to the nature of physics material which obligates using math and diagrams to enable the student understand and solve problems.

In addition, teachers still suffer from model-developing skills which prevent them from relating mathematical examples to scientific models. Moreover, such a result might also be referred to lack of technical teaching aids in schools as that contributes to lack of practicing computational thinking.

By analyzing results of supervisors, it was noted that they all confirmed that physics teachers practice mathematical thinking during the teaching process, but to a small extent. Few of them assign their students scientific tasks whose solutions need math and mathematical thinking. As for computational thinking, the supervisors also pointed out that its use and practice were lacking as well. They attributed that to several reasons among which are: lack of teacher preparing programs, low level of awareness, shortage of equipment and computer sets in some schools, besides teaching and administration loads assigned for them, and for large number of students. They indicated that solving such a problem is important and can be achieved through providing teachers, who practice this activity, with moral and material incentives.

Such a result disagrees with those of the following studies: (Harris et al., 2017 ; Tuttle et al., 2016 ; Brownstein & Horvath, 2016 ; Daisly, 2016 ; Kawasaki, 2015), which revealed a satisfactory level in the performance of physics teachers with regard to NGSS. But such a result agrees with results of the following studies: (Hang & Srisawasdi, 2021 ; Alshyab, 2019 ; Aboathrah, 2019) which indicated that this practice ranked "low".

- **Constructing interpretations and solution design**

Table 7 presents results pertaining this practice

Table 7. Means and standard deviations for the degree of availability regarding the practice of constructing and designing solutions and their indications.

No.	Items	Mean	Standard deviation	Degree of availability	Rank

1-	I request students to adopt authentic and correct evidence to explain certain physical phenomena.	2.59	1.34	Weak	4
2-	I request them to design various physical projects.	2.02	0.77	Weak	7
3-	I request them to apply scientific law.	2.71	1.17	Medium	3
4-	I request them to apply ideas of physics to construct interpretations for similarities and differences between things or different phenomena.	2.57	1.34	Weak	5
5-	I request them to construct scientific interpretations based on evidences to show how certain external factors affect a physical phenomenon	2.73	1.32	Medium	2
6-	I request them to apply principles and scientific theories to design a way to reduce the influence of certain factors on various physical phenomena.	2.20	0.96	Weak	6
7-	I request students to construct a scientific interpretation based on evidences that describe how differences in certain features increase the possibility of survival of the physical phenomenon.	2.79	1.22	Medium	1
	**General mean**	**2.52**	**0.51**	**Weak**	

Table 7 shows that the general mean for the availability of this practice ranked "weak", (2.52), while three items ranked "medium" (no.7) rated first, followed by (no. 5) second, and (no. 3) third. Such results reflect that teachers are still below the expected level regarding student’s need to possess scientific evidence to present his ideas or explain physical phenomena. The rest of items ranked "weak".

Although physics obligates the student to make scientific comparisons based on knowledge or scientific laws, yet teaching this subject still lacks the practices needed to achieve such a goal, because traditional methods of teaching are still being used.

In this respect, result of this study disagrees with those of the following: (Harris et al., 2017 ; Daisly, 2016 ; Kawasaki, 2015), whose results regarding the issue ranked "medium". While it is agreed with the study (Hang &

Srisawasdi, 2021; Alshyab, 2019; Aboathrah, 2019), which showed a medium and low degree of science teachers' practice of constructing interpretations and designing solutions.

Analyzing supervisors’ interviews revealed that the viewpoints of two of them agree with questionnaire results: teachers practice data interpretation and solution design to a great extent in teaching; they assign students tasks to design solutions for scientific problems.

Other five supervisors pointed out that the activity was not that much practiced. They attributed that to insufficient time set for class which doesn’t help the teacher to do the practice, besides the shortage of teachers assigned for the practice. The supervisors added that the clearly stated designs and solutions found in teachers’ guide are overlooked by them, but they never try to apply them in class. Thus, teaching physics remains traditional which solely depends on memorization. Such opinions agree with results of the following studies: (Alshyab, 2019 ; Aboathrah, 2019).

- **Involvement with pretexts, evidences, and proofs**

Table 8. presents results pertaining this issue

Table 8. Means and standard deviations for the degree of availability regarding the practice of involvement with pretexts, evidences, and proofs and their indications.

No.	Items	Mean	Standard deviation	Degree of availability	Rank
1-	I encourage students to present assessments and justifications for constructing physical models and scientific explanations.	2.83	1.05	Medium	2
2-	I request them to use the pretext supported by evidence and experiment to clarify different physical systems	2.59	1.19	Weak	5
3-	I request them to compare different models and interpretations that disclose points of strength and weakness	2.63	1.25	Medium	4
4-	I request them to use verbal and written pretexts to support or refute a model or an interpretation of a certain physical phenomenon	2.69	1.06	Medium	3
5-	I encourage them to explain evaluations and	2.84	1.12	Medium	1

evidences for each other.				
**General mean**	**2.72**	**0.68**	**Medium**	

Table 8 shows that the general mean for the availability of this practice, ranked "medium", (2.72), while (14) items ranked "medium" except for (no. 2) which ranked "weak".

One can notice that teachers still suffer from this practice, so some of them try to avert it by not asking students to evaluate or explain any phenomenon. Teaching and administration loads also add up to the suffering of teachers. Therefore, they still need training in the domain of constructing models and in conveying that to their students.

Results of supervisors' interview agree, to an extent, with those of the questionnaire which revealed an acceptable degree of this practice that was manifested in providing evidences in presenting scientific knowledge, even via the traditional method, though most of that is done theoretically. The practical and applicable pretexts were rarely used because of lack of suitable equipment and labs in schools, in addition to short class time, large number of students, and burdens of teaching and administration.

Such a result agrees with those of the following studies. (Alshyab, 2019 ; Malkawi & Rababah, 2018 ; Aboathrah, 2019; Harris et al., 2017 ; Tuttle et al., 2016 ; Daisly, 2016 ; Kawasaki, 2015) which revealed that this practice ranked "medium" in some items of teachers' performance.

- **Obtaining, evaluating, and communicating information.**

Table 9 presents results pertaining this issue

Table 9. Means and standard deviations for the degree of availability regarding the practice of Obtaining, evaluating, and communicating information and their indications.

No.	Items	Mean	Standard deviation	Degree of availability	Rank
1-	I request students to distinguish between observation and induction, request and evidence, pretext and interpretation	3.14	0.74	Medium	4
2-	I request them to use diverse means of communication patterns such as: diagrams, models,	3.71	0.76	High	1

	and equations.				
3-	I request them to read scientific physical texts and interpret them	2.45	1.32	Weak	5
4-	I request them to produce a scientific text in order to develop and explain a physical model	3.20	0.71	Medium	3
5-	I request students to collect data in order to describe a certain physical phenomenon	3.24	0.75	Medium	2
	**General mean**	**3.15**	**0.33**	**Medium**	

Table 9 shows that the general mean for the availability of this practice was "medium", (3.15). item (no.2) rated first ranking "very high". Such a result might be attributed to the nature of physics which necessitates using diagrams and scientific laws. Three items ranked "medium" (no. 5) rated second, (no. 4), third, and (no. 1) fourth, while (no. 3) rated last, "weak".

This indicates that teachers might not have considered that gaining and evaluating information are two essential segments of scientific work. Therefore, teachers of physics need to be convinced that the student should specify the suitable authentic source for his data and information, besides specifying the suitable mechanism to deal with such data to benefit from them in teaching or in real life. They also need to read scientific texts and encourage students to do that, silently or loudly. Students as well should concentrate on all basic scientific and integrated processes to secure education situations that enable them to practice the process clearly.

Such a result agrees with those of the following studies: (Hang & Srisawasdi, 2021 ; Alshyab, 2019 ; Aboathrah, 2019 ; Qablan, 2016) which pointed out that teachers' performance of this practice ranked "medium". But this result disagrees with those of the following studies: (Harris et al., 2017 ; Tuttle et al., 2016 ; Daisly, 2016 ; Kawasaki, 2015) which revealed the existence of a suitable level in teachers' performance regarding NGSS.

By analyzing results of supervisors, it was noted that they all confirmed that a number of teachers never deviate from the textbook as the source of knowledge. They attributed that to administrative and technical reasons associated with time, lack of good training, and technical labs in schools. In addition, some of them don't know how to deal with sources of technical information. Yet, they pointed out that teachers request students to read ready-scientific texts, but they rarely ask students to invent or produce scientific texts for the sake of designing a scientific

model, or explaining a certain phenomenon. Supervisors' opinion agree with what the following studies came up to. (Alshyab, 2019 ; Hang & Srisawasdi, 2021 ; Aboathrah, 2019 ; Qablan, 2016).

Results of the second questions: " Does that degree of availability in performance of those teachers vary in accordance with qualification, or years of experience?"

Qualification

"t" test was conducted to detect the significant differences between means of teachers' results pertaining qualification variable. Table 10 illustrates that.

Table 10. (t) test results to detect statistical differences between means of physics teachers' responses pertaining qualification variable.

Qualification	N	mean	Std. Deviation	t	df	Sig.
Bachelor's	31	2.65	0.23	3.59	47	0.001
Postgraduate	18	2.88	018			

Table 10 reveals that there are significant difference between means in favor of postgraduate teachers whose higher studies tackled modern strategies of teaching, international and national standards that include NGSS and that was well reflected in their performance. In addition, programs of higher studies in universities care about modern trends in teaching, the most significant of which at present is NGSS. Such results agree with that of (Malkawi & Rababah, 2018).

Years of experiences

One Way- ANOVA was used to analyze significant differences between means of teachers' results in accordance with years of experience variable as presented in table 11.

Table 11. One Way – ANOVA for comparing means of physics teachers responses in light of years of experience variable.

Variance source	Sum of Squares	Mean Square	df	f	Sig.
Between Groups	0.22	0.11	2	1.97	0.151
Within Groups	2.593	0.056	46		
Total	2.815		48		

Table 11 shows that there are no differences with statistical significance between the two means that might be attributed to teachers' updated knowledge regarding NGSS, engineering and scientific practices pertaining difference in years of experience, or lack of in-service training associated with such standards and practices. Such results agree with those of the following studies: (Alshyab, 2019 ; Malkawi & Rababah, 2018) which revealed that there were no differences with statistical significance that might be attributed to years of experience with regard to science teachers pertaining engineering and scientific practice at the secondary stage.

Conclusion

As stated in the introduction, to implement NGSS, there is an urgent need to conduct studies on the scientific and engineering practices in teaching physics (NRC, 2012). The purpose of this study is to detect the degree of availability of eight engineering and scientific practices in the performance of physics teachers from their own perspective and the perspective of education supervisors.

Although the study was limited to the interview of (6) education supervisors, yet it took into consideration the viewpoints of the majority of physics teachers. The viewpoints of the two groups, teachers and supervisors, were concordant to a great extent.

The results revealed that teachers might not have understood or knew all scientific and engineering practices with their indications which were expected to be perfected by the students. Therefore, they were not manifested as required in their performance. Hence, teachers need sufficient support which enables them to completely understand them in a way that complies with NGSS so as to eventually be able to help their students understand them. This support can be achieved when the Ministry of Education takes into consideration such practices and indications. The Ministry also needs to train teachers and to reconsider courses offered by faculties of education in the domain of preparing physics teachers and to relate that to NGSS. There is also a need to practically apply such practices throughout pre-and in-service training of teachers.

Funding:

The researcher is grateful to Taif University, represented by the Deanship of Scientific Research, for funding this study. Research project number (34-442-1).

Declarations

Conflict of interest The author declare that he have no conflicts of interest.

Ethical Approval: Scientific Research Ethics Approval was obtained from the Scientific Research Ethics Committee at Taif University, No.43-024

References

Aboathrah, S. (2019)The level of practice of physics teachers for the secondary stage of the next generation of science standards. *Journal of Umm Al-Qura University for Educational and Psychological Sciences, 10*(2), 100-134, https://drive.uqu.edu.sa/_/jep/files/10-2/jep-10-2-2-2.pdf

Achieve (2013*). Next Generation Science Standards: Adoption and Implementation. Washington*, DC: The U.S. Education Delivery Institute. Retrieved on: 10/05/2021 at*:* http://www.nextgenscience.org/standards-background-research-and-reports

Alebous, T. (2021). The level of understanding scientific and engineering practices in light of the next generation science standards among preservice student teachers. *Educational Research and Reviews, 16*(3), 51-63. https://doi.org/10.5897/ERR2020.4111

Alshyab, M. (2019). Possession Level Of Scientific And Engineering Practices In The Light Of The Next Generation Science Standards (NGSS) By Secondary Stage Science Teachers In Saudi Arabia. *Journal of Umm Al-Qura University for Educational and Psychological Sciences, 10*(2), 338-366. https://drive.uqu.edu.sa/_/jep/files/10-2/jep-10-2-2-10.pdf

Bielik, T., Finnie, K., Peek-Brown, D., Klager, C., Touitou, I., Schneider, B. & Krajcik, J. (2021). High School Teachers' Perspectives on Shifting towards Teaching NGSS-Aligned Project Based Learning Curricular Units, *Journal of Science Teacher Education*. https://doi-org.sdl.idm.oclc.org/10.1080/1046560X.2021.1961973

Boesdorfer , S. & Staude, K. (2016). Teachers' Practices in High School Chemistry Just Prior to the Adoption of the Next Generation Science Standards, *School Science and Mathematics,116*(8), 442-458. https://doi.org/10.1111/ssm.12199

Brownstein, E. & Horvath, L. (2016). Next Generation Science Standards and edTPA: Evidence of Science and Engineering Practices. *Electronic Journal of Science Education, 20* (4), 44-62.

Bybee, R. (2014). NGSS and the next generation of science teachers. *Journal of Science Teacher Education*, *25*(2), 211–221. https://doi.org/10.1007/s10972-014-9381-4

Daisly, P. (2016). *The Next Generation Science standards: Understanding High School Teachers Perspectives on Implementation*. Doctorate thesis, Washington State University.

Dalvi, T., Mangiante, E., & Wendell, K. (2021). Identifying Pre-service Teachers' Conceptions about the NGSS Practices Using a Curriculum Critique and Revision (CCR) Task, *Journal of Science Teacher Education, 32*(2), 123-147. https://doi-org.sdl.idm.oclc.org/10.1080/1046560X.2020.1791465

Duschl, R., & Bybee, R. (2014). Planning and carrying out investigations: An entry to learning and to teacher professional development around NGSS science and engineering practices. *International Journal of STEM Education*, *1*(1), 1-9. https://doi.org/10.1186/s40594-014-0012-6.

Fulcher, T. (2014). *Supplemental Curricula and Science and Engineering Practices in the Next Generation Science Standards: Developing a Tool for Identification and Alignment*. Master thesis, University of Wyoming

Harris, K. & Sithole, A. & Kibirige, J. (2017). A Needs Assessment for the Adoption of Next Generation Science Standards (NGSS) in K-12 Education in the United States. *Journal of education and training studies, 5*(9), 54-62. https://doi.org/10.11114/jets.v5i9.2576

Jimenez-Liso, M., Chico, M., Avraamidou, L., & Villegas, R. (2021). Scientific practices in teacher education: the interplay of sense, sensors, and emotions, *Research in Science & Technological Education*, 39(1), 44-67. https://doi: 10.1080/02635143.2019.1647158.

Kawasaki, j. (2015). *Examining teachers' goals classroom instruction around the science and engineering practices in the next generation science standards*. Unpublished dissertation university of California.

Kawasaki, J., & William, S. (2020). Examining teachers' classroom strategies to understand their goals for student learning around the science practices in the Next Generation Science Standards. *Journal of Science Teacher Education. 31*(4), 384-400. https://doi.org/10.1080/1046560X.2019.1709726

National Research Council (2012). *A Framework for K-12 Science Education: Practices, Crosscutting Concepts, and Core Ideas. Committee on a Conceptual Framework for New K-12 Science Education Standards (Board on Science Education, Division of Behavioral and Social Sciences and Education)*. Washington, DC: The National Academies Press.

NGSS Lead States. (2013). *Next Generation Science standard: for states*. By states. Washington: The National Academies Press.

Hang, N., & Srisawasdi, N., (2021). Perception of the Next Generation Science Standard instructional practices among Vietnamese pre-service and in-service teachers, *Journal of Technology and Science Education*, 11(2), 440-456. http://dx.doi.org/10.3926/jotse.1154

Malkawi, A., & Rababah, E., (2018). Jordanian twelfth-grade science teachers' self-reported usage of science and engineering practices in the next generation science standards. *International Journal of Science Education, 40*(9), 961-976. https://doi.org/10.1080/09500693.2018.1460695

Pruitt, S, (2015). The Next Generation science standard: where are we now, and have we learned? Retrieved on: 1/05/2021 at *https://static.nsta.org/pdfs/samples/tst1505_17.pdf*.

Qablan, A. (2016). Teaching and Learning about Science Practices: Insights and Challenges in Professional Development. *Teacher Development Journal. 20* (1), 76–91. https://doi.org/10.1080/13664530.2015.1111929

Tyler, B., & DiRanna, K. (2018). *Next Generation Science Standards in practice: Tools and processes used by the California NGSS Early Implementers*. San Francisco, CA: WestEd.

Severance, S., Penuel, W., Sumner, T., & Leary, H. (2016). Organizing for teacher agency in curricular co-design. *Journal of the Learning Sciences, 25*(4), 531–564. https://doi.org/10.1080/10508406.2016.1207541

Stuart, Z., Kelly, M., Westerfeld, D., & Bugallo,M. (2021). NGSS Engineering Practices in Physics Instruction: Building a Night Light. *The Physics Teacher, 59*(3), 206-209. https://doi.org/10.1119/10.0003668

Tuttle, N. & Kaderavek, J. & Molitor, S. & Czerniak, C. (2016). Investigating the Impact of NGSS-Aligned Professional Development on PreK-3 Teachers' Science Content Knowledge and Pedagogy. *Journal of Science Teacher Education*, *27*(7), 717-754.

Zhang, X., & Wong, J. (2021). How do teachers perceive their knowledge development through engaging in school-based learning activities? A case study in China. *Journal of Education for Teaching International research and pedagogy*. *48*(1), 1-19. https://doi.org/10.1080/02607476.2021.1947135
