# Supplementary material for: Scientific and engineering practices aligned with the NGSS in the performance of secondary stage physics teachers
Source: PLoS One. 2022 Oct 10;17(10):e0275158. doi: 10.1371/journal.pone.0275158 (PMC9550033; doi:10.1371/journal.pone.0275158)
Supplement: Attachment — Submitted filename: Coment on PLOS ONE review research.pdf [file pone.0275158.s006.pdf]

Title:

This title is vague. I could not identify the research objective through this title; is it an analysis of teachers' observations, an analysis of teacher programs, or just a survey study?

I think the study deals with the extent to which teachers adopt scientific and engineering practices in their teaching from their point of view.

Aligning means that the study analyzed teacher programs based on teachers' actual practices. Therefore, this study has nothing to do with aligning

Language:

All manuscript needs proof reading.

Abstract:

The method is not clear in the abstract.

Introduction:

There are no paragraphs related to the objective of the study

Questions:

The method is not clear in the abstract

Method:

Description of the instruments, the way of calculated the values related to the criterion of judgement such as intervals and therefore, this affected the assignment of the levels in all tables.

Results:

The criterion of judgement was as follows

degree of accessibility?

I think it is the level or degree of practice from their point of view.

Discussion:

The discussion is too short. You should use a separate section for the discussion and make it richer and more organized. Too short and does not do justice to the study's richness.

References:

Minor corrections are needed for the references.
