# Supplementary material for: Scientific and engineering practices aligned with the NGSS in the performance of secondary stage physics teachers
Source: PLoS One. 2022 Oct 10;17(10):e0275158. doi: 10.1371/journal.pone.0275158 (PMC9550033; doi:10.1371/journal.pone.0275158)
Supplement: Attachment — Submitted filename: Response to Reviewers.docx [file pone.0275158.s004.docx]

Dear reviewers,

The study was proofread by a specialized proofreader. The main terms of the study have been revised, the language checked, and several changes made to ensure lucidity and comprehensibility. Technically, the study was checked by a specialist in statistical analysis to verify the soundness of the analysis conducted throughout. All the remarks provided by the reviewers have been addressed below.

Reviewer # 1

| Response | Remark |
| --- | --- |
| Modified. The paragraph was rephrased. | Overview/Statement of the Problem/Significance - The sentence "Many institutions catered for developing the field of teaching and learning science, relying on such standards" does not have a clear meaning and should be rewritten to clarify the subject, verb, and object of the sentence. |
| Modified. Clarified the first time it was used. | Early in the manuscript, the authors need to clarify that NGSS stands for "Next Generation Science Standards." |
| Modified. A number of studies were cited to corroborate the author’s assertions. | - The introductory paragraph consists mainly of the authors' assertions without a strong evidentiary base. If an overview of the standards is warranted here, it should be grounded in evidence-based claims from reputable sources. |
| The theoretical background on the NGSS was expanded and supported. However, it is not clear what the reviewer means in this remark when saying “regarding copyright and trademark.” Further explanation is needed for the author to be able to address this issue. | - The citation for the Next Generation Science Standards documents does not match the information provided on the NGSS website itself regarding copyright and trademark: <https://www.nextgenscience.org/trademark-and-copyright> |
| Modified. Author’s assertions have been backed up by previous research including the example provided. | - Assertions such as "Because students’ objectives for learning sciences in general and physics in particular have changed, science teachers of all levels need to adapt themselves to these new standards in order to achieve an actual change in their teaching practices. Therefore, educators see that teachers’ qualifying programs should be developed to make teachers concentrate more on actual practice and to help them amend their practices." (p. 2) appear throughout the manuscript and lack references and citations to support them. Because of this pattern, many of the premises that follow from these assumptions appear unfounded. |
| Modified. This sentence was misphrased as a goal of the NGSS. It has been phrased correctly as a claim by previous research which has been cited. | - Despite claims by the authors, a goal of the NGSS "to make students behave and practice in a scholarly manner to gain skills of engineering design which enable them to conduct research and to solve problems they encounter throughout science study or in their actual life" appears nowhere in the standards documents. For reference: https://www.nextgenscience.org/faqs |
| Modified. The practices have been rephrased bearing in mind gender-neutrality and font issues as described in the comment here. | - The Eight Science and Engineering practices described on page 3 are written in gender-biased language (deictically referring to "student" as "he/him/his." Further, typeface choices such as setting certain words in boldface are distracting to the reviewer. |
| Modified.  The Introduction section has been renamed “Introduction and Literature Review” In this section, the topic of the study is introduced along with reviewing previous research on the topic. This is why no separate section for (literature review) was created. | Review of Literature - There is no clear section that reviews literature; therefore, it is unclear how the current manuscript is building on or extending prior work in this field. |
| Modified. A description of “mixed approach” has been added to the “Study Approach and Participants” section. | Methodology - While the study refers to a "mixed approach" to research, the author should clarify whether this was a mixed methods design (and if so, how), or whether there were simply multiple methods or multiple sources of data. |
| Modified. The consent procedures in the “Study Approach and Participants” section now explains that the consent was a written one. | - Consent procedures should specify whether oral or written consent was provided by participants. |
| 1- More details were added to the “Study Limitations” section.  2- More details about the sampling of the participants were added to the “Study Approach and Participants” section  3- More details were added in the “Questionnaire” and “Interview” sections to explain how validity and reliability were tested and verified.  4- The qualitative trustworthiness has been elaborated, and the whole “Interview” section was modified. | 1- Study limitations are not discussed with sufficient detail.  2- A much clearer explication of the sampling frame is necessary to evaluate the procedures of random and purposive selection.  3- A check for validity was referenced, although there is no clear discussion of whether this is for construct, content, face, or criterion validity. Further, despite the mentioning of an exploratory survey, no statistical calculations or data are provided to support the authors claims of validity and reliability.  4- In the case of qualitative trustworthiness, there is no mention whatsoever of credibility, transferability, dependability, confirmability, or authenticity. |
| The metric is provided in the “Questionnaire” section. It is as follows: “The questionnaire responses were judged according to the following criterion: if the mean is (1.80) or less, the degree of presence is “very weak”; (more than 1.80 -2.60) “weak”; (more than 2.60-3.40) “medium”; (more than 3.40-4.20) “high”; and (more than 4.20) “very high.” | - There are no clearly grounded definitions for the metric of "degree of availability," and thus, it appears to be an arbitrary construction of the authors. If any rating scale is to be used, it should be one that is grounded in prior evidence and tested for validity and reliability through established statistical analysis. |
| Modified. A separate paragraph was added explaining how the qualitative data was analyzed. | - There is no clear discussion of how qualitative interview data was analyzed, nor of how the conclusions were reached. |
| Since the above statistical issues have been addressed, the ANOVA should deliver reliable results. In addition, the statistical analysis of this study was presented to a specialist in educational statistics who approved of the reliability of the ANOVA test results in this study. | - Given the above statistical issues, the ANOVA does not appear to offer valid or reliable results. |

Reviewer # 2

| Response | Remark |
| --- | --- |
| Modified. Vancouver Style used. | 1. PLOS uses “Vancouver” style, as outlined in the ICMJE sample references. Please modify it. |
| The placement of the “Study Limits and Limitations” section before the “Results” section is in keeping with a convention in research writing. If the reviewer is giving a choice of replacing this section in the “Conclusion,” the author would like to keep it as is. It is worth mentioning that the study’s limits and limitations were restated in the second paragraph of the “Conclusion” section. | 2. I suggest that the author move the section "Study limits and limitations" to the paragraph "Conclusion". |
| Modified. More details about the questionnaire were added in the “Questionnaire” section to address this issue. | 3. The source of the basis for the evaluation criteria of the questionnaire explored? |
| Yes. They were based on the same literature from which the eight scientific and engineering practices were derived. The “Interview” section has been modified to clarify this point. | 4. Do these eight open-ended questions have relevant literature basis? |
| The demographics of the participants were mentioned in the “Study Questions” section (question 2) and they were elaborated in the discussion of the results. | 5. The problem of demographic variables was not seen in the research. |
| Added. The explanation of why the F test was used in this study was added in the “Results” section when addressing the “years of experience” variable. | 6. The teacher’s seniority is a continuous variable. Why the F test is used in this study, please add the author's explanation. |

Reviewer # 3

| Response | Remark |
| --- | --- |
| Modified. Problem statement added. | 1. There is no problem statement. |
| The study used a closed-ended questionnaire. The type was added to the “Abstract” section and the “Questionnaire” section. | 2. It is not stated what questionnaire was used. |
| Modified. Implications were stated in the abstract. | 3. The implications of the findings are not clearly stated in the abstract |
| Modified. Acronym explained. | Introduction 1. NGSS is the first word mentioned on page 1, but it is an acronym and it is not shown what it stands for. |
| This section has been renamed “Introduction and Literature Review” based on one of the remarks of Reviewer # 1. Therefore, it ended up being a lengthy section because it tackles two parts, i.e. introducing the topic and reviewing the relevant literature. | 2. The introduction and theoretical background is rather long. The format of having subtopics “significance of the study, terms of the study are from the dissertation format. |
| The NGSS build on the previous standards. They are a progression from these standards. This relationship is stated in the transitions between the first and the second paragraphs of the “Introduction” section. | 3. There are many standards mentioned such as NSTA, NSF, AAAS but what is the purpose for and how is it different from the NGSS? What is the change and adaptation that needs to be done? This needs to be more specific. |
| The 8 practices were clearly referenced at the beginning of the paragraph where they are presented.  More literature has been added to substantiate these practices. | 4. There were 8 practices outlined on page 3. These practices should be refenced. And literature can be added to build upon it as it seems to form the theoretical background of the study. |
| The sentence has been rephrased as the following: “Educators and those involved in the NGSS believe that these standards in general should be present in the performance of physics teachers.” | 5. On page 4: “Educators and those concerned with NGSS see that physics teachers should possess such standards in general” Do teachers “possess standards? |
| Modified. The phrasing of the study’s title, problems, and questions has been changed.  So, the question now reads, “What is the degree to which scientific and engineering practices aligned with the NGSS are present in the performance of physics teachers at the secondary stage? | 6. In the research questions there is mention of degree of availability ie “What is the degree of availability pertaining scientific and engineering practices that cope with NGSS in the performance of physics teachers at the secondary stage? What exactly does this term mean? |
| No, there isn’t. | 7. Is there any hypothesis for the study. |
| Modified. This section was rewritten, and the mixed method was explained and referenced. | 8. On page 5, it was stated that this is a mixed method approach. Is there a reference for the framework of this mixed method study? |
| The participants are teachers at the secondary stage in addition to educational supervisors. In Saudi Arabia’s educational system, educational supervisors are in charged with monitoring the performance of teachers and providing feedback to them. | 9. Who exactly are the participants? If they are teachers, why do they need supervisors? Or are these preservice teachers? |
| a. Modified according to Vancouver Style  b. Modified. The validity of the instrument has been elaborated. | 10. On page 6, it was written: “After determining objectives of the questionnaire, the researcher checked the official website of NGSS (https:www.nextgenescience.org), modern education literatures, besides surveying scientific and engineering practices relevant to teachers of physics at the secondary stage as presented in (Malkawi & Rababah, 2018 ; Hang & Srisawasdi, 2021 ; Alshyab, 2019 ; Aboathrah, 2019 ; Brownstein & Horvath, 2016 ; Qablan, 2016 ; Fulcher, 2014 ; Stuart et al., 2021 ; Duschl & Bybee, 2014 ; Kawasaki, 2015), and .. a. The writing of in-text citation above is incorrect. b. The validation of the instrument is also not clear. |
| It is not clear what is required here. An example is provided, but it is not explained what is wrong with it.  In this study, the eight scientific and engineering practices were formulated as the main items of the questionnaire and were assigned a number of questions. The responses to the questions pertaining to each practice were presented in a separate table and analyzed afterwards. This is considered an acceptable convention of presenting and analyzing data in research writing. | 11. The presentation of the findings is not well done. eg. on page 8: The item of teachers’ scientific and engineering practices might be elaborated on as follows: •Asking questions and defining problems Table 2. presents results pertaining this type of practice. |
| Yes. The t-test is the correct means of analysis for this question. This test was chosen based on the consultation of a specialist in statistical analysis. | 12. The second RQ on page 19: Does that degree of availability in performance of those teachers vary in accordance with qualification, or years of experience?", was analysed using t-tests. However, the variables seem to be of nominal and ration data respectively. Is t-test the correct means of analysis. |
| Modified. Specific recommendations have been added. | 13. What are the implications to the study. And the recommendations should be made more specifically |
