# Supplementary material for: Scientific and engineering practices aligned with the NGSS in the performance of secondary stage physics teachers
Source: PLoS One. 2022 Oct 10;17(10):e0275158. doi: 10.1371/journal.pone.0275158 (PMC9550033; doi:10.1371/journal.pone.0275158)
Supplement: Attachment — Submitted filename: renamed_5e89d.docx [file pone.0275158.s007.docx]

The content has been amended to better reflect the work conducted.

The author has made a clear description of the source of the questionnaire.
