# Supplementary material for: Scientific and engineering practices aligned with the NGSS in the performance of secondary stage physics teachers
Source: PLoS One. 2022 Oct 10;17(10):e0275158. doi: 10.1371/journal.pone.0275158 (PMC9550033; doi:10.1371/journal.pone.0275158)
Supplement: Attachment — Submitted filename: Reviewer reports.docx [file pone.0275158.s008.docx]

Reviewer reports:

It's my pleasure to review the manuscript titled " Scientific and engineering practices aligned with the NGSS in the performance of secondary stage physics teachers ", and I appreciate the research idea.

However, there are some points need to be addressed; especially, in relation to the research methodology and discussion. Thereby, some suggestions are given below for improving the quality of the manuscript.

Point 1: In the (Abstract): researcher confirmed that there are two aims for this study which were not appear in the literature review. At the end of the (Introduction and Literature Review): researcher stated that (….this study attempts to unveil the extent to which the NGSS-aligned scientific and engineering practices are present in the performance of physics teachers of the secondary stage). Therefore, research needs to modify the literature to include all the aims of this study and questions.

Point 2: under the (Study Problem): (…However, despite the latest reforms related to involving students in physics, there are still big challenges that confront teachers who attempt to constantly apply science practices in class [32]…) What are these reforms and big challenges? I think this sentence need to be rewritten and clarified more.

Point 3: The whole section of (Significance of the Study) needs to be deleted, and no need for it in the structure of writing this article.

Point 4: The whole section of (Terms of the Study) needs to be deleted and some the information in relation to the definitions just need to be added to the literature review.

Point 5: The whole section of (Study Limits and Limitations) needs to be moved to different parts in this article. First: researcher needs to clarify which one of the three major dimensions of the NGSS was chosen and why. This needs to be explained in the literature review. Second: the limitations regarding the sample of the study need to be moved to the end of the article under the limitations section.

Point 6: under the (Methodology): researcher did not specify which one of the mixed methods approaches was applied in this study. which one did the researcher start with it before the other (questionnaire or interviews)?.....

Point 7: under the (Study Instruments): the researcher stated that (….. After determining the objectives of the questionnaire, the scientific and engineering practices for physics teachers at the secondary stage were identified from the official website of the NGSS …). What are these objectives and on which basis these practices were identified?

Point 8: In terms of the validation of the questionnaire, researcher applied the internal consistency (Correlation coefficient) which I do not think this is the correct statistical method for the validation of the questionnaire. I believe that researcher should apply the Factor analysis and revise the items of the questionnaire with experts in the same field for validation; especially, that the NGSS was built in different environment.

Point 9: Under the (Study Result): all the results need to be separated from the discussion and need to be shortened. The section of discussion needs to cover all the results from quantitative and qualitative data with deep conversation. Researcher has done quite discussion through connecting the result from the questionnaire with interviews and comparison with the result of the other studies. However, this is not enough to explore the reasons stand behind the results reported in this study.

Point 10: Under the (Conclusion): Conclusion needs to be rewritten again. it should be a summary for only the ideas that have strong finial impression. Also, all the limitations under the conclusion need to be moved to the new section named Limitations. In addition, all the information from (…the results revealed that the teachers did...) needs to be placed under new section called Implications of this study.

Finally, there is a need to bring uniformity in the reference section.
