# Supplementary material for: Scientific and engineering practices aligned with the NGSS in the performance of secondary stage physics teachers
Source: PLoS One. 2022 Oct 10;17(10):e0275158. doi: 10.1371/journal.pone.0275158 (PMC9550033; doi:10.1371/journal.pone.0275158)
Supplement: Attachment — Submitted filename: Response to Reviewers2222222.docx [file pone.0275158.s009.docx]

Dear reviewers,

The study was proofread by a specialized proofreader. The main terms of the study have been revised, the language checked, and several changes made to ensure lucidity and comprehensibility. Technically, the study was checked by a specialist in statistical analysis to verify the soundness of the analysis conducted throughout. All the remarks provided by the reviewers have been addressed below.

Reviewer # 3

| Response | Remark |
| --- | --- |
| Modified | 1. Abstract does not indicate the need for the study. |
| The problem is clear | 2. On page 2. The problem statement is not clear. You have mentioned that science teachers of all levels need …………. |
| It has been shown that teachers' practices contribute to the development of standards in students | 3. On page 3, you have stated the practices in NGSS…………. |
| Modified | 4. On page 4, first paragraph, it is stated: “Because previous studies ………….. |
| Modified | 5. On page 4, the research questions refer to the “degree of availability”. This is not defined. The research …………… |
| The importance section has been removed as requested by reviewers | 6. In the significance of the study, it was mentioned that methods of assessment of teachers could be beneficial for ………….. |
| It has not been deleted, due to the importance of defining the study terms | 7. On page 5, the terms of the study are stated. This reads like a thesis, and not like a journal article. |
| Because the study focuses on physics teachers in the city of Taif only | 8. The study was limited to a sample from Taif city. What is the rationale for selecting teachers in Taif, ……….. |
| Because the website is the primary reference for NGSS standards | 9. The NGSS website was mentioned several times. Why was the website used and not an official document? |
| The questionnaire was built by the researcher after referring to the references and studies | 10. The questionnaire that was used for the study (page 6), is it a new questionnaire designed for the study or one which is already available? |
| This is explained in the construction and application procedure of the interview | 11. When interviews were used, it would be valuable to see the interview protocols and what was asked…………. |
| Modified | 12. The limitations of the study needs to be mentioned. This would enable suggestions for futre studies. |
| The manuscript was revised by an English language specialist | The article needs to be proofread to ensure the language is of quality |

Reviewer # 5

| Response | Remark |
| --- | --- |
| The study attempted to reveal the degree of availability of scientific and engineering practices aligning with NGSS standards in the performance of teachers | **Title:**  This title is vague. I could not identify the research objective through this title; is it an analysis of teachers' observations…………… |
| The manuscript was revised by an English language specialist | **Language:**  All manuscript needs proof reading. |
| Modified | **Abstract:**  The method is not clear in the abstract. |
| Modified | **Introduction:**  There are no paragraphs related to the objective of the study |
| Modified | **Questions:**  The method is not clear in the abstract |
| Modified | **Method:**  Description of the instruments, the way of calculated the values …………. |
| Yes | **Results:**  The criterion of judgement was as follows  degree of accessibility? ……… |
| Since the manuscript presented the results of each scientific and engineering practice separately, this requires the presentation and discussion of each result directly. | **Discussion:**  The discussion is too short. You should use a separate section for the discussion and make it richer and more organized. ………. |
| Modified | **References:**  Minor corrections are needed for the references. |

Reviewer # 6

| Response | Remark |
| --- | --- |
| The questionnaire was built by the researcher after referring to the references and studies | Comment 1: I am sorry to say that this manuscript is confusing and, at times, was disjointed and unclear. …………….. |
| Ok | Comment 2: Simply stated, the author of this study, based on the NGSS website and several studies, identified 8 variabl…… |
| It was clarified that the exploratory sample was outside the study sample | Comment 3: Then, an exploratory sample of 15 physics teachers was used to provide validity and reliability statistics…………. |
| Modified | Comment 4: Then, this instrument was completed by 49 randomly selected ………………. |
|  | Comment 5: Then, each teacher's total score on all 8 variables in this instrument was used to identify ………………… |
| This classification was adopted after referring to several studies that used it | Comment 6: Then, the teachers were divided into three groups based on their reported years of experience ……………. |
| This was clarified, and that supervisors give their opinions based on their actual follow-up of these teachers | Comment 7: Finally, 6 teachers' supervisors were picked and interviewed to …………... |
| The characteristics of the psychometric instruments have been confirmed | Comment 8: Clearly, this instrument is central to this study, and therefore, all the rules regarding test construction and statistical matrices apply.….. |
| Modified | Comment 9: The structure and the presentation of the manuscript are much more consistent with what is ……….. |
| Modified | Comment 11: Many paragraphs before methodology are unnecessary or can be trimmed to one or two sentences. For example, the study problem ……………….. |
| Modified | Comment 12: Reading paragraph 4 on pages 2 and 3 gives the impression that the author constructed ……………. |
| Modified | Comment 13: Everything before the methodology needs to be re-written under the introduction ………….. |
| Modified | Comment 14: The presentation of results needs improvements. The table should be formatted differently………. |
| Since scientific and engineering practices are the focus of this study, it is better to present each practice in a separate table, as well as discuss it independently | Comment 15: It is unclear why a table for each practice (variable) is shown. Such a presentation might ………….. |
| Since the manuscript presented the results of each scientific and engineering practice separately, this requires the presentation and discussion of each result directly. | Comment 16: Also, the author does not seem to distinguish …………… |
| Modified | Comment 17: The limitations section needs to be well thought out and more clearly written and articulated |

Reviewer # 7

| Response | Remark |
| --- | --- |
| Modified | 1. Introduction and literature review : first paragraph (1-4) who? “ explained that education ….)………….. |
| Modified | 2. The study limits section to be transferred to the end of discussion |
| Yes | 3. The results and discussion parts are mixed. |
| Modified | 4. Table 3 showed that general mean stated as weak while in the text stated as medium. |

Reviewer # 8

| Response | Remark |
| --- | --- |
| In the literature of the study, all the aims of the study were addressed | Point 1: In the (Abstract): researcher confirmed that there are two aims for this study which were …………………. |
| modified | Point 2: under the (Study Problem): (…However, ………….. |
| modified | Point 3: The whole section of (Significance of the Study) needs to be deleted, and no need for it in the structure of writing this article. |
| It has not been deleted, due to the importance of defining the study terms | Point 4: The whole section of (Terms of the Study) needs to be deleted and some the information in relation to the definitions just need to be added to the literature review. |
| modified | Point 5: The whole section of (Study Limits and Limitations) needs to be moved to different parts in this………… |
| It was mentioned that the questionnaire was applied to the teachers first, then the interview to the supervisors | Point 6: under the (Methodology): researcher did not specify which one of the mixed methods.......... |
| The objectives of the questionnaire are the same as the objectives of the study, and they were predetermined | Point 7: under the (Study Instruments): the researcher stated that (….. After determining the …………….. |
| A doctor specializing in educational statistics was consulted, and he stated that the internal consistency is very appropriate | Point 8: In terms of the validation of the questionnaire, researcher applied the internal consistency ……….. |
| Since the manuscript presented the results of each scientific and engineering practice separately, this requires the presentation and discussion of each result directly. | Point 9: Under the (Study Result): all the results need to be separated from the discussion and need to be ……….. |
| modified | Point 10: Under the (Conclusion): Conclusion needs to be rewritten again. it should be a summary for only ………... |
| Verified | Finally, there is a need to bring uniformity in the reference section. |
