# Supplementary material for: Scientific and engineering practices aligned with the NGSS in the performance of secondary stage physics teachers
Source: PLoS One. 2022 Oct 10;17(10):e0275158. doi: 10.1371/journal.pone.0275158 (PMC9550033; doi:10.1371/journal.pone.0275158)
Supplement: Attachment — Submitted filename: Response to Reviewers.docx [file pone.0275158.s010.docx]

Reviewer # 6

| Response | Remark |
| --- | --- |
| Modified: The results have been separated from the discussion | I am sorry that the author has not adequately addressed…………. |
| Modified: The results have been separated from the discussion | Overall, there need to be significant …………… |

Reviewer # 7

| Response | Remark |
| --- | --- |
| The study was proofread by a specialized proofreader | The first paragraph in introduction need punctuation, the paper need language editing before publishing. |
| Modified: The results have been separated from the discussion | the result and discussion sections should be separated and not mixe |

Reviewer # 8

| Response | Remark |
| --- | --- |
| Modified | Point 1: In terms of the aims of the study that were mentioned in the abstract…… |
| Modified | Point 2: under the (Methodology): researcher still ……………. |
| Modified | Point 3: Under the (Study Limits and Limitations), researcher did not clarify which one of the three major dimensions of the NGSS ………. |
| Modified | Finally: Under the (Conclusion): there were no modifications that were made by ………. |
