# Supplementary material for: Scientific and engineering practices aligned with the NGSS in the performance of secondary stage physics teachers
Source: PLoS One. 2022 Oct 10;17(10):e0275158. doi: 10.1371/journal.pone.0275158 (PMC9550033; doi:10.1371/journal.pone.0275158)
Supplement: Attachment — Submitted filename: Response to Reviewers.docx [file pone.0275158.s011.docx]

Reviewer #6:

| Firstly, the eight practices and the 44 indicators are not separate dimensions……………… | Modified |
| --- | --- |
| Secondly and most importantly, the issue of reliability and validity. As I have indicated in my first review of this manuscript, a serious issue exists in establishing the validity and reliability of this instrument ………. | Modified |

Reviewer #8:

| Reviewer #8: For the manuscript titled " Scientific and engineering practices aligned with the NGSS in the performance of secondary stage physics teachers ", there still some limitations under the section of “Conclusion”. All these limitations under the conclusion need to be moved to the “study limits and limitations” and make them concise. | Modified |
| --- | --- |
